# Review of field and monitoring studies investigating the role of nitro-substituted neonicotinoid insecticides in the reported losses of honey bee colonies (*Apis mellifera*)

**DOI:** 10.1007/s10646-016-1734-7

**Published:** 2016-10-05

**Authors:** Richard Schmuck, Gavin Lewis

**Affiliations:** 1 Bayer CropScience AG, Monheim am Rhein, Germany; 2 JSC International Ltd., Harrogate, UK

**Keywords:** Neonicotinoids, Risk assessment, Bee monitoring, Honey bees, Bumble bees, Solitary bees

## Abstract

The nitro-substituted neonicotinoid insecticides, which include imidacloprid, thiamethoxam and clothianidin, are widely used to control a range of important agricultural pests both by foliar applications and also as seed dressings and by soil application. Since they exhibit systemic properties, exposure of bees may occur as a result of residues present in the nectar and/or pollen of seed- or soil-treated crop plants and so they have been the subject of much debate about whether they cause adverse effects in pollinating insects under field conditions. Due to these perceived concerns, the use of the three neonicotinoids imidacloprid, clothianidin and thiamethoxam has been temporarily suspended in the European Union for seed treatment, soil application and foliar treatment in crops attractive to bees. Monitoring data from a number of countries are available to assess the presence of neonicotinoid residues in honey bee samples and possible impacts at the colony level and these are reviewed here together with a number of field studies which have looked at the impact of clothiandin on honey bees in relation to specific crop use and in particular with oilseed rape. Currently there is considerable uncertainty with regards to the regulatory testing requirements for field studies. Accordingly, a testing protocol was developed to address any acute and chronic risks from oilseed rape seeds containing a coating with 10 g clothianidin and 2 g beta-cyfluthrin per kg seeds (Elado^®^) for managed honey bee (*Apis mellifera*) colonies, commercially bred bumble bee (*Bombus terrestris*) colonies and red mason bees (*Osmia bicornis*) as a representative solitary bee species. This is described here together with a summary of the results obtained as an introduction to the study details given in the following papers in this issue.

## Introduction

The nitro-substituted neonicotinoid insecticides, which include imidacloprid, thiamethoxam and clothianidin, are an agronomically very important group of insecticides which are widely used to control a range of important agricultural pests both by foliar applications and also as seed dressings and by soil application. They are agonists of insect nicotinic acetylcholine receptors (nAChR) and, therefore, often effectively control pest species which have evolved resistance to other insecticide classes like acetylcholinesterase inhibitors (organophosphates, carbamates) and sodium channel agonists (pyrethroids). Since neonicotinoids show systemic activity, exposure of bees may occur as a result of residues present in the nectar and/or pollen of seed- or soil-treated crop plants or when applied as a spray before flowering. Due to their high intrinsic toxicity to honey bees, nitro-substituted neonicotinoid insecticides have been intensively examined worldwide by regulatory agencies for their risks posed to honey bees. In Europe, the active substance clothianidin whose impact to bees were further investigated as outlined in the following chapters was included in Annex I to Directive 91/414/EEC on 1 August 2006 by Commission Directive 2006/41/EC.

In spring 2008, a number of bee poisoning incidents were reported during sowing of seed-treated maize in the Upper Rhine valley and in parts of Bavaria in Germany (Pistorius et al. 2009). The reason for the poisoning was the abrasion of dust from maize seeds treated with the insecticide Poncho Pro (a.s. clothianidin) and subsequently during the sowing process with pneumatic sowing machines, the blowing out of this dust containing the active substance into the environment. Most probably, forager bees passively collected these dust particles and returned a mix of dust and pollen back to their hives. Due to this incident, the specific provisions of the approval were amended by Commission Directive 2010/21/EU, to permit use of clothianidin, and other nitro-substituted neonicotinoids (thiamethoxam and imidacloprid) as well as the insecticide fipronil (an antagonist of GABA- and glutamate-gated chloride channels that is also highly bee-toxic) as seed treatments only where the seed coating is performed in professional seed treatment facilities. These must apply the best available techniques to ensure that the release of dust during application to the seed, storage and transport can be minimised, and where adequate drilling equipment is used to ensure a high degree of incorporation in soil resulting in a minimisation of dust emission.

In the years following the incident in the Upper Rhine valley, a large number of scientific investigations were published with a special emphasis on the sub-lethal effects of nitro-substituted neonicotinoids on bees (e.g., Whitehorn et al. 2012; Henry et al. 2009). The European Commission asked the European Food Safety Authority (EFSA) for scientific and technical assistance to assess this new information and to review the risk assessment of clothianidin (and the other nitro-substituted neonicotinoid active substances imidacloprid and thiamethoxam) regarding the impact of the currently authorised uses of these substances as seed treatments and granules on bees. EFSA published its conclusions on the risk assessment for clothianidin (revised) on 14 March 2013 (EFSA 2013a). As for thiamethoxam (EFSA 2013b) and imidacloprid (EFSA 2013c), high acute risks for bees were alleged for plant protection products containing clothianidin from exposure via dust from the drilling of several crops, from consumption of residues contained in pollen and nectar for some seed-treated crops and from exposure via guttation fluid. Furthermore, the EFSA identified a number of data gaps for each of the evaluated compounds, particularly focusing on long term risk to honey bees from exposure to dust, residues in pollen and nectar, and guttation fluid. Concerns were also raised for bee species other than domestic honeybees.

In the light of this evaluation, the Commission considered that the approved uses of clothianidin, thiamethoxam and imidacloprid may no longer satisfy the approval criteria provided for in Regulation (EC) No 1107/2009 with respect to their impact on bees and that the perceived high risk for bees could not be excluded except by imposing further restrictions. Accordingly, Commission Implementing Regulation (EU) No 485/2013 amended the conditions of inclusion of the active substances clothianidin, thiamethoxam and imidacloprid, by limiting the use of plant protection products containing those active substances to professional uses. Further, uses as seed treatment and soil treatment of plant protection products containing clothianidin, thiamethoxam or imidacloprid were prohibited for crops attractive to bees except for uses in greenhouses. Foliar treatments with plant protection products containing clothianidin, thiamethoxam or imidacloprid were prohibited for crops attractive to bees with the exception of uses in greenhouses, uses in crops which are harvested before flowering and uses after flowering.

## Evidence of effects on honey bee colonies under agricultural conditions

### Observations on the development of managed bee colonies

Numerous studies have reported a decline in honey bee health and numbers of colonies in recent years (e.g., Potts et al. 2010; Van Engelsdorp et al. 2007) but there are other voices which challenge the contention that overwintering losses are any worse now than they have been in the past (Doebler 2000; Borst). Bee keepers in a number of countries have reported a decline in the ability of colonies to successfully survive the winter. A very extreme form of bee decline was reported from the USA with a sudden disappearance of all but a few bees from managed bee colonies and this phenomenon was called Colony Collapse Disorder (Van Engelsdorp et al. 2008). In Europe, a similar phenomenon has not been observed and larger numbers of colony deaths have generally been locally confined (e.g., Genersch et al. 2010). In recent times, the most dramatic decline in managed colonies in Europe occurred in the 1990’s coinciding with the socio-political changes in Eastern Europe while at the same time colony numbers remained stable in Western Europe (Moritz et al. 2010). Here, colony losses have been more varied over time and between countries as shown by Hendrikx et al. (2012) when looking at overwintering losses from 2000 to 2009 in Denmark, Finland, Sweden, Germany, England and Wales. A baseline colony loss rate of around 10 % was identified but peaks of over 30 % were seen in some countries in certain years. In general, colony losses have been compensated for by beekeepers replacing colonies as particularly seen in the 70’s and 80’s in response to the appearance of *Varroa destructor*, resulting in a steady increase in the number of managed colonies overall (Moritz et al. 2010).

Many factors may have contributed to a decline in honey bee health and increases in bee or even colony losses, where they occur. The spread of parasites and pathogens is a possible cause, although no single agent has been identified and instead a virulent combination of parasites and pathogens has been suggested (Chen and Evans 2007; Johnson et al. 2009). Other possible factors include a reduction in available forage (Decoutye et al. 2010), beekeeping management practices (for example insufficient *Varroa* control and the development of resistance to treatments), movement of colonies, weather and climate change (Kluser et al. 2011). Exposure to pesticides is another factor that has been implicated in bee health decline (Mullin et al. 2010). Although the majority of cases where bees are killed by pesticides are caused by foliar-applied products (Fletcher and Barnett 2003; Barnett et al. 2007; Thompson and Thorbahn 2009), the current public discussion is focused on systemic seed treatment products. It is proposed by some researchers that adverse effects may occur when bees are feeding on seed-treated crops or dust drift-contaminated plants or if they collect contaminated pollen and/or nectar from these plants and return it to the hive. However, major incidents related to seed treatments with nitro-substituted neonicotinoid insecticides have only been related to the emission of dust during drilling of corn seeds which had been improperly treated (Pistorius et al. 2009). Dust emission during maize drilling has also been discussed as a potentially contributing factor of spring bee mortality in Italy (Greatti et al. 2002). However, some of the reported changes in colony numbers pre-date the agricultural use of neonicotinoids and so it is important to look at the overall picture as well as identifying specific information linking possible neonicotinoid exposure to observed colony losses.

### Findings from monitoring studies and field surveys

Monitoring data from a number of countries are available to assess the presence of neonicotinoid residues in honey bee samples and possible impacts of these residues at the colony level. A review of winter losses of bee colonies up to 2008 under the auspices of the German Bee Monitoring Project is provided by Janke and Rosenkranz (2009). This was carried out by establishing a database of 120 apiaries and 1200 bee colonies over a period of four years. Data were collected for a range of parameters including for the apiary (e.g., site, nuclei, movement of colonies, *Varroa* treatment), strength of the colonies in autumn and spring, honey yields, residues in bee bread (stored pollen) and bee disease analysis. Overwintering losses of the monitored colonies ranged between an average of 7.9 and 15.9 % over the four project years. Of all apiaries participating during the four project years, nearly one-third had no losses while about 15 % had losses over 20 %. Bee bread samples collected during or after the flowering of oilseed rape in spring were analysed using a sensitive multi-residue method. In 215 analysed samples collected from 2005 to 2007, clothianidin was not found in any sample while imidacloprid was found in only one sample (3 μg/kg). Some samples contained no residues but the majority did and in most cases more than one active ingredient was found with at least 55 being identified overall (usually only in trace amounts). Nearly 4400 data sets were statistically analysed for the identification of triggers with negative influence on overwintering. The winter losses were significantly correlated with *Varroa* infestations and virus infections in autumn. It was concluded that no acute effects of pesticides on honey bee colonies were expected on the basis of the evaluated residue data but additional work was suggested to further assess long-term effects on e.g. colony development and over-wintering success.

In a follow-up paper, Genersch et al. (2010) provided a more detailed assessment of the German Bee Monitoring Project. All data were statistically analysed in respect to the overwintering mortality of the colonies. It was demonstrated for several factors that they are significantly related to the observed winter losses of the monitored honey bee colonies as previously described, i.e. high *Varroa* infestation level and virus infection (specifically deformed wing virus (DWV) and acute bee paralysis virus (ABPV)) in autumn. A clear significant effect was shown for the age of the queen: colonies that survived the winter had on average significantly younger queens compared to the colonies that collapsed during winter. A clear effect on overwintering success was also found for colony strength (number of bees) in October. There was no significant difference in overwintering success between apiaries with no pesticide residues in the bee bread and those with higher amounts of residues. In addition, there was no significant correlation between winter losses and the amount of oilseed rape pollen in the honey harvested in summer (in Germany, oilseed rape seed was almost 100 % treated with neonicotinoid insecticides during the monitoring period). Some residues were identified in bee bread although mainly substances which are considered non-toxic for bees and the observed amounts of the residues were quite low i.e., three orders of magnitude lower than the respective LD_50_ values. Accordingly, no relation between chemical residues in pollen and colony development or winter losses could be demonstrated even though particular emphasis was put on this aspect.

Foraging honey bee, honey and pollen trap samples collected from eighteen apiaries in Western France (Bretagne and Pays de la Loire) from four different landscape contexts during four different periods in 2008 and in 2009 were analysed to evaluate the presence of pesticides and veterinary drug residues (Lambert et al. 2013). A multi-residue analysis was developed to identify and quantify 80 pesticides (covering the majority of active ingredients used for plant protection) and veterinary drugs in the three beehive matrices. A total of 141 honey bee, 141 honey and 128 pollen samples were collected from the 18 apiaries during 2008 and 2009. Of these, 102 (72.3 %), 135 (95.7 %) and 75 (58.6 %) of the samples, respectively, contained at least one of the defined 80 chemicals. The frequency of detection was higher in the honey samples (28 compounds) than in the pollen (23 compounds) or honey bee (20 compounds) samples, but the highest concentrations were found in pollen. Although most compounds were rarely found, some of them reached high concentrations that might lead to adverse effects on bee health. The three most frequent residues in all three matrices were the widely used fungicide carbendazim and two acaricides, amitraz and coumaphos, that are used by beekeepers to control *Varroa destructor*. Clothianidin was not found at all while imidacloprid was only detected in 3 out of 141 honey samples (2.1 %) and in 1 of the 128 pollen samples (0.8 %) and was not found among the 141 honey bee samples. While it was recognised that lower levels established for this monitoring exercise could be present (e.g., <LOD for clothianidin in pollen of 1.4 µg/kg), the study authors noted that no adverse effects had been identified under laboratory conditions at the detection limits used in this study.

In Greece, sudden deaths, unusual behaviour and declines of adult honey bee populations were reported in summer 2009 by the beekeepers in the region of Peloponnese, Greece (Bacandritsos et al. 2010). A preliminary study was carried out to investigate these unexplained phenomena in this region and samples were collected from the affected colonies. The clinical symptoms of selected colonies were noted and 37 bee samples were collected from five different apiaries suffering similar symptoms. Symptomatic adult honey bees tested positive for *Varroa destructor, Nosema ceranae*, chronic bee paralysis virus (CBPV), acute paralysis virus (ABPV), deformed wing virus (DWV), sacbrood virus (SBV) and black queen cell virus (BQCV). Chemical analysis revealed that amitraz, thiamethoxam, clothianidin and acetamiprid were all absent although imidacloprid was present in three out of the five apiaries sampled. Residue levels were detected at an average concentration of 27 µg/kg tissue which is equivalent to a dose of about 3 ng/bee. This residue level is close to the lower threshold of the calculated acute oral LD_50_ dose which range between 3.7 and 40.9 ng/bee (Schmuck et al. 2001). However, the presence of multiple pathogens and pesticides made it impossible to associate a single specific cause to the depopulation phenomena observed in Greece. The authors identified five possible factors: (i) multiple virus infection by five different viruses along with infection by *N. ceranae,* (ii) imidacloprid residues in bee tissues, (iii) stress induced by transportation, (iv) temperature and humidity fluctuations and (v) the collection of fir honeydew (low water content).

In another study carried out in Greece (2011–2013), honey bee, bee pollen and honey samples from different areas across the country where incidents had been reported were analysed for the presence of pesticide residues (Kasiotis et al. 2014). In the majority of cases areas were rural with substantial agricultural activity. Honey bee samples were collected very near to or at the entrance of the hives, while bee pollen was collected from the hives. In total, 71 samples of honey bees, bee pollen and honey were collected by individual beekeepers or other authorities (e.g., veterinary institutes) usually after the report of honey bee death incidents during 2011, 2012 and 2013 (44 honey bees, 14 bee pollen and 13 honey samples). From the analysis of the samples the presence of 14 active substances was observed in all matrices, with 73 % of honey bees, 43 % of pollen and 0.1 % of honey samples being positive for at least one compound. A number of neonicotinoids were identified, including clothianidin, which had concentrations in honey bees ranging from 0.7 to 14.7 ng/bee in 2011, 2.7 to 39.9 ng/bee in 2012 and 6.1 to 6.8 ng/bee in 2013 i.e. around the acute lethal oral dose. Since the sample sizes in terms of numbers of analysed bees were very small, e.g., only 17 honey bees in 2011, 19 in 2012 and 8 in 2013, the available data tend to indicate that locally high levels of residues may occur but they do not appear to be widespread and cannot be responsible for any general trends.

In Italy, two regions in the North (Lombardy and Veneto) organised an institutional network whereby if beekeepers observed damage to their bees they were requested to report it to the local Veterinary Authority and fill in a questionnaire (Bortolotti et al. 2009). This was followed up with inspection of the apiaries and the collection of samples (dead bees and pollen from surrounding vegetation) for analysis of pathogens and neonicotinoid residues. Collected data indicate that the higher number of bee loss events occurred in intensively cultivated flat areas, located in the North of Italy, mainly during or after maize sowing. The chemical analyses of dead bees (105 samples) revealed the presence of three neonicotinoid residues: imidacloprid was found in 25.7 % of the samples, thiamethoxam in 2.8 %, clothianidin in 25.7 %, both imidacloprid and thiamethoxam in 4.7 %. The concentrations ranged from 1.01 to 240.6 ng imidacloprid/g, from 3.67 to 39.2 ng clothianidin/g and from 24.8 to 138 ng thiamethoxam/g. Following visual examination and virological analyses the authors did not consider pathological causes as being responsible for the reported damages. They concluded that the spatial and temporal correlation between reported bee mortality and maize sowing and the presence of residues of active ingredients used for seed dressing (imidacloprid, thiamethoxam and clothianidin) in almost half of the samples confirmed a correlation between bee mortality and the sowing of corn seed dressed with neonicotinoids. The use of these neonicotinoids for seed dressing of maize was therefore suspended in Italy in September 2008. Following this, monitoring was carried out from March to May 2009 in 60 hives belonging to 10 different apiaries spread across representative areas of the Friuli Venezia Giulia region (Frilli et al. 2009). It was concluded that no population decline or mortality occurred in the hives in 2009 at the time of maize-sowing.

In Canada, data obtained from the Canadian Pest Management Regulatory Agency (PMRA) was reviewed by Cutler et al. (2014a). Since 2007, 110 honey bee-pesticide incident reports were received by the PMRA but there were very few incidents (six) reported up to 2011. However, in 2012 a significant number of incidents were reported in the province of Ontario and Quebec, where exposure to neonicotinoid dust during planting of corn was suspected to have caused the incidents in about 70 % of cases (either alone or in combination with other pesticides). Most of these incidents were classified as ‘minor’ by the PMRA, and only six cases were considered ‘moderate’ or ‘major’, involving 402 colonies. In that same year, however, there were over three times as many moderate or major incidents due to older non-neonicotinoid pesticides (mainly dimethoate or chlorpyrifos), involving 3855 colonies, i.e., moderate or major incidents due to non-neonicotinoid pesticides affected nearly 10× more hives than incidents related to neonicotinoid pesticides. These data thus agree with the general conclusion with regards to seed dust, i.e., exposure of honey bees to neonicotinoid-containing dust during corn planting needs to continue to be mitigated. However, even in this specific case it identifies the need for the risk assessment to be carried out in a balanced manner for a sustainable agroecosystem, taking into account that other pesticides may pose an even greater risk.

In contrast to maize (corn), there has been no evidence that planting canola (oilseed rape) seed treated with neonicotinoid insecticides in Canada places pollinators at risk. Seed treatments used for canola have a very limited potential to release dust particles, and field studies investigating exposure of honeybees to systemic residues in pollen and nectar of seed-treated canola show no chronic or acute poisoning when analysed at field scale rates (Cutler and Scott-Dupree 2007; Cutler et al. 2014b). In the past decade, the number of honey bees in Canada has reached near-record levels, i.e., more than 700,000 colonies Canada-wide in 2012, up from 600,000 in 2000 (Statistics Canada Cansim Table 001–007). More than 70 per cent of these colonies are in Western Canada, where canola (which is largely seed-treated with nitrosubstituted neonicotinoids) has become one of the most important crops. Clothianidin was first registered in Canada as a seed treatment on canola at the end of 2003 and so its use has been contemporaneous with this increase in colony number i.e. there are no indications of any adverse effects.

Overall then, the available monitoring data indicates that there is no widespread correlation between neonicotinoids such as clothianidin (either in terms of use or residues present in bee matrices) and observed trends in honey bee performance, either in general or in terms of colony losses. The exceptions to this were the cases of an inadequate seed treatment quality of maize seed drilled in the Upper Rhine valley in Germany in 2008 and the correlation between mortality and maize sowing in Italy (together with the presence of residues of active ingredients used for seed dressing). Also, in spring 2011 a high number of bee poisoning incidents was recorded during the sowing of maize in the Pomurje region of Slovenia and the presence of clothianidin in dead bees and pollen was attributed to the sowing of maize treated with the insecticide Poncho Pro (Van der Geest 2012). However, the specific issue of maize seed treatment can be addressed by appropriate risk management measures relating to improvements in seed treatment quality and drilling technology. Apart from this, the only other occurrence of high levels of residues that may be linked to effects on honey bees is localised (related to specific circumstances, e.g., crop, application and local conditions) and does not appear to be responsible for any general trends.

This conclusion is further supported by preliminary results on a survey about bee colony mortality of the 2014/15 season which were published by the independent bee researchers’ network COLOSS. In total, 23,234 beekeepers contributed data to the survey, representing a total of 469,249 honey bee colonies. Respondents are from 31 countries (most EU and a few non-EU countries in Europe and the Mediterranean Region). Losses were particularly heterogeneously distributed between countries as well as between regions. High losses seem to have occurred in particular in the countries of the central region of Europe, but an obvious or consistent pattern is not recognizable: colony loss rates per country varied from 5 % (Norway) to 36 % (Belgium). Overall loss rate in the evaluated countries was 17.4 %, which is twice as high as in the previous year. More details can be found under http://www.coloss.org/announcements/losses-of-honey-bee-colonies-over-the-2014-15-winter-preliminary-results-from-an-international-study. There was a particularly low loss rate immediately after the 2013/14 season when the full spectrum of neonicotinoid products were still on the market and before the restriction came into force, but twice as high a loss rate in the next season (2014/15) when the restrictions were in place. This indicates that the restrictions of the neonicotinoids in the EU did not lead to an immediate improvement of bee health, and further suggests that there is no correlation between colony losses and the use of neonicotinoids. Nevertheless, there is not a tendency towards a generally improving bee health situation in Europe and loss rates are varying according to patterns which are not yet fully understood from year to year and from region to region.

## Findings from field studies using a highly bee attractive seed-treated crop (oilseed rape)

A number of studies have looked at the impact of clothianidin on honey bees in relation to specific crop use and in particular with oilseed rape. A long-term investigation was conducted in Canada to assess effects on honey bee colonies during and after exposure to flowering oilseed rape (canola) grown from clothianidin treated seed (Cutler and Scott-Dupree 2007). Honey bee colonies were placed in the middle of 1 ha fields: four treated and four control fields with four colonies in each field. In the treated fields, the oilseed rape seed was treated with Poncho 600 FS (a.s. clothianidin) at 400 g a.s./100 kg seed and sown at 8.0 kg/ha so that clothianidin was applied at 32 g a.s./ha. Bee mortality, worker longevity and brood development were regularly assessed in each colony over 130 days (3 weeks of flowering followed by a post-exposure period at a holding apiary). Samples of honey, beeswax, pollen and nectar were regularly sampled over this period for analysis of clothianidin residues. Overall, no differences in bee mortality, worker longevity, or brood development occurred between control and treatment groups throughout the study. Weight gains and honey yields from colonies in treated fields were not significantly different from those in control fields. Only 14 % of colonies alive in autumn did not survive winter (considered typical for the region) and half of those were control colonies. Although clothianidin residues were detected in honey, nectar, and pollen from colonies in clothianidin-treated fields and in a few control nectar samples, maximum concentrations detected were 8- to 22-fold below the reported NOEC of 20 ppb. Clothianidin residues were not detected in any beeswax sample. It was concluded that field exposure to clothianidin seed-treated oilseed rape poses negligible risk to honey bees.

A second study was initiated in 2012 in a large-scale field experiment in southern Ontario, Canada, to determine whether exposure to clothianidin seed-treated oilseed rape has any adverse impacts on honey bees (Cutler et al. 2014b). Four colonies were placed in each of five clothianidin seed-treated or five control canola fields during flowering. The seed was treated with a formulation containing 20.4 % clothianidin at a rate of 1.4 L/100 kg seed, which was sown at a rate of 5.6 kg seed/ha. After flowering, the colonies were moved to an apiary with no surrounding crops grown from seeds treated with neonicotinoids. Colony weight gain, honey production, pest incidence, adult mortality, number of adults, and amount of brood were assessed in each colony throughout summer and autumn. Samples of honey, beeswax, pollen, and nectar were regularly collected, and were analysed for clothianidin residues. Assessments were also made in spring 2013: determination of dead and live colonies; capped brood assessment; adult strength assessment; determination of presence of queen, eggs and larvae; beeswax for residue analysis; and pest incidence. Overall, colonies were vigorous during and after the exposure period, and no effects of exposure to clothianidin seed-treated oilseed rape on any assessment endpoints were found. Bees foraged heavily on the test fields during peak flowering and residue analysis indicated that honey bees were exposed to low levels (0.5–2 ppb) in pollen. Overwintering success did not differ significantly between treatment and control hives, and was similar to overwintering colony loss rates reported for the winter of 2012–2013 for beekeepers in Ontario and Canada. Again it was concluded that exposure to canola grown from seed treated with clothianidin poses low risk to honey bees.

Consistent residue data were obtained from a field study in which two fungicides (boscalid and prothioconazole) were sprayed onto an 8 ha field of flowering oilseed rape grown from clothianidin treated seed in accordance with normal agricultural practice in Germany (Wallner 2009). Over a 7-day period, residues were measured in pollen and nectar loads of returning foragers. Clothianidin was neither detected in the pollen loads of returning bees over the whole period nor in pollen or honey. Clothianidin was detected in the nectar in the honey sac loads over the 7-day period, with levels ranging between 1 and 3 ng/g i.e. close to the limit of quantitation.

In Poland, honey bee colonies were sampled in an intensively cultivated area of oilseed rape treated with neonicotinoid insecticides (Pohorecka et al. 2012). Clothianidin, thiamethoxam or imidacloprid were used as seed treatments and acetamiprid or thiacloprid were used as foliar sprays. Fifteen healthy bee colonies were placed in close proximity to each of the oilseed rape fields and samples of nectar and pollen were collected throughout the flowering period and samples of bee bread, honey and adult bees were taken one week after the end of flowering. All the applied neonicotinoid insecticides were present in the nectar and pollen samples although they were at levels well below the acute oral and contact LD_50_ values (Blacquière et al. 2012). Thiamethoxam, thiacloprid and acetamiprid were the most frequently detected while clothianidin was found in only 17 % of the combined nectar and honey samples (average content of 2.3 ng/g) and in 11 % of the pollen samples (average content of 1.8 ng/g). However, while the risk of exposure of bee colonies to pesticide residues was considered relatively high in areas of oilseed rape no negative effects of neonicotinoids on the bee mortality, brood development, strength and honey yield of healthy bee colonies were found throughout the study period.

More recently, work has been carried out in Sweden to investigate how neonicotinoids influence bees, including wild species, in real-world agricultural landscapes when applied to spring oilseed rape (Rundlöf et al. 2015). The study was set up using replicated and matched landscapes, with 8 pairs of summer oilseed rape fields (mean size of 8.9 ha and separated by at least 4 km) located across southern Sweden. One field in each pair was sown with spring oilseed rape seeds treated with 25 mL/kg Elado (400 g/L clothiandin and 80 g/L β-cyfluthrin) and the fungicide thiram while the control seeds were treated with thiram only. The sowing rate was 7.7 and 7.5 kg/ha, respectively. At each field, estimates were made of: (1) the density of wild bees; (2) the nesting activity of the solitary bee *Osmia bicornis*; (3) the colony development of the bumble bee *Bombus terrestris*; and (4) the strength of honey bee colonies. It should be noted that the residue levels in nectar and pollen of seed-treated spring oilseed rape reported by the authors are significantly higher (6.6–23 and 6.7–16 ng/g in honey bee collected pollen and nectar, respectively) than found in a study on winter oil seed rape (maximum levels of 1.6 and 1.1 ng/g, respectively), which were considered to be consistent with other studies (see Rolke et al. 2016b). The study authors concluded that the seed treatment of spring oilseed rape with clothianidin reduced the density of wild bees (bumble bees and solitary bees) in the flowering oilseed rape fields and adjacent uncultivated field borders. There was also a reduction in the nesting of the solitary bee on the treated fields, which was linked to the ability of emerging females to build brood cells. However, solitary bee populations are not homogeneously distributed in the landscape and overall numbers found in the study were very low (less than 50 bees in total in each of the treated and control groups). The main wild bees observed during transect walks belong to groups of ground nesting species highly adapted to a specific nesting resource, which might be sparse in an agricultural landscape and no female of *Osmia bicornis* were observed foraging on the oilseed rape. The hatching of both males and females at almost the same time (males might appear 1–3 days earlier than females) is critical to ensure the mating in very small populations and if this does not occur it could lead to a higher dispersal of individuals. In addition, no assessment of nesting females was performed so that it is not clear how many of the hatched females would have started to build a nest.

Six commercial bumble bee (*Bombus terrestris*) colonies were placed at each of the test fields in Sweden and the insecticide seed coating was also negatively related to colony growth and reproduction of the bumble bee. The rate of weight gain of colonies at the treated fields and the number of queens and worker/male cocoons produced at the end of the season was lower than the control fields. However, reproductive parameters in bumble bees are very variable and the number of colonies placed at each site was low and also non-standardized hives were used. It should also be noted that indoxacarb, which can be toxic to bumble bees for up to two days after application, was sprayed in one treatment replicate on the same day as bumble bee hives had been placed in the field (see also Sterk et al. 2016).

The other main finding of the study was that the insecticide treated seeds had no significant influence on honey bee colony strength. Thus, while the results for honey bees are consistent with other studies (e.g., Genersch et al. 2010; Pohorecka et al. 2012; Cutler et al. 2014b; Rolke et al. 2016b), it was concluded by the authors that such insecticidal use may pose a risk to wild bees in agricultural landscapes. However it is difficult to interpret the data on the absolute number of individuals, as information for species distribution on the individual plots is not available. In addition, the individual abundances recorded were too low to substantiate a robust conclusion about potential effects. For bumble bees, only two generalist species showed differences in abundance in total between control and treatment while specialist and more rare species showed little or no difference.

## Large-scale monitoring study to further address risk uncertainties raised by EFSA

The available field and monitoring data for assessing the possible effects of seed treatments with nitrosubstituted neonicotinoids of a bee attractive crop like oilseed rape, generally indicate no effects on honey bee colonies and this is associated with low levels of residues in relevant crop matrices (Schmuck et al. 2001; Cutler and Scott-Dupree 2007,2014; Pilling et al. 2013). Nevertheless, the EFSA concluded after reviewing all available data that acute and chronic risks to honey bees cannot be excluded (EFSA 2013a). The EFSA risk assessment and resulting conclusion was mainly based on laboratory data or studies on individual honey bees since all submitted data from studies conducted under more realistic bee exposure conditions were considered to be supplementary information only and not sufficient to revise the basic Tier 1 risk conclusion. The key rationale provided for excluding higher tier studies from the risk assessment was that the statistical power of these higher tier studies was not sufficient as they were either non-replicated or not sufficiently replicated. However, in such circumstances it is possible to use a weight of evidence approach, taking into account all available information e.g., from single location, non-replicated studies and monitoring programs, to arrive at a comprehensive and conclusive risk evaluation (Van Der Kraak et al. 2014). Furthermore, EFSA concluded that in the case of wild bees (e.g., bumble bees and solitary bees), there is less data available (e.g*.,* Cutler and Scott-Dupree 2014) and the results are more variable so it is not currently possible to determine if there are effects from the use of clothianidin as a seed treatment.

Currently, there is considerable uncertainty about the regulatory risk assessment for bees in the EU and in particular with regards to the regulatory testing requirements for field studies. A number of initiatives have been established but the process will take considerable time and therefore, does not provide timely guidance to notifiers as to how any uncertainties identified should be addressed using higher tier testing approaches. Due to the importance of sustaining crop protection solutions against soil pests in oilseed rape, an advanced field testing protocol was developed to address the concerns of EFSA (see Heimbach et al. 2016). This testing protocol was used for the study reported in the following papers and was designed to deliver reproducible results with a high statistical power for appropriately addressing any acute and chronic risks:

1. to managed honey bee (*Apis mellifera*) colonies (see Rolke et al. 2016a)
2. to commercially bred bumble bee (*Bombus terrestris*) colonies (see Sterk et al. 2016)
3. to mason bees (*Osmia bicornis*) as a surrogate species for solitary bee species (see Peters et al. 2016)

It is important to note though that there is currently a poor understanding of the population level (colony) biology of bumble bees and solitary bees so it is difficult to put current information into a realistic context. Therefore, the study reported here attempted to address this challenge by appropriately integrating existing experiences from commercial use of bumble bees and mason bees into the study design.

Currently available field studies tend to follow the principle of exposing the examined species to a single source of exposure, i.e., a crop field, which serves either as a control or test site. Little attention is paid to the surrounding landscape and associated stress factors, which may influence the development of the examined species (Pilling et al. 2013; Cutler et al. 2014b; Cutler and Scott-Dupree 2014; Garbuzov et al. 2015). In replicated field studies, treatments other than the product under investigation are often not controlled and may differ between control and test sites or between replicates (e.g., Rundlöf et al. 2015). Accordingly, the large-scale field monitoring study protocol outlined in the paper of Heimbach et al. (2016) aimed to minimize these methodological shortcomings and to comply as much as possible with the requirements of the draft bee guidance document of the European Food Safety Authority.

The chosen test design consisted of two large circular areas of approximately 65 km² each (diameter 9 km) in north-east Germany, which were very similar in terms of climatic and pedological/geological conditions. The dominant crop at both sites was winter oilseed rape, with a total of 614.6 ha constituting 16.0 % of the core area at the reference site (7 km diameter), and 791.7 ha (20.6 %) at the test site. No other mass flowering crop that could have been suitable as bee forage and could have influenced the development of the exposed bee species were present during oil seed rape flowering. The scale of these landscapes ensured that in terms of agricultural land, the introduced bee species only foraged either on winter oilseed rape or on uncropped headlands which contained a few wild plant species in flower but did not receive any chemical treatment. The bees will also have foraged to varying degrees in off-crop habitats e.g., hedges, kettles, and forest edges. However, the exposure of bees to chemical stressors during the study was largely the same between the test and reference sites except for the exposure to systemic clothianidin residues in the oil seed rape fields of the treated area. Thus, at the reference site, study fields were drilled with clothianidin-free oilseed rape seeds while at the test site the oil seed rape seeds contained a coating with 10 g clothianidin and 2 g beta-cyfluthrin per kg seeds (Elado^®^). For winter oilseed rape, the study represented a worst case exposure situation to bees since the local, oilseed rape cultivation practice resulted in a maximum duration of exposure. The large size and number of fields (17 and 18 fields with median sizes of 33.5 and 35.3 ha for the reference and test fields, respectively) required the drilling of different varieties, i.e., early and late flowering varieties to allow more time for harvesting. This mix of varieties with different times of flowering also resulted in a prolonged overall flowering period for the seed-treated winter oilseed rape and, thus, in the maximum length of exposure for the introduced bee species.

Inevitably, given the nature and scale of the study some variation between the sites will have occurred. Both sites were located in traditional areas of oilseed rape cultivation and each area contained more than 600 ha of oilseed rape, which required efficient pest control to safeguard the harvest. Accordingly, the pest control by the clothianidin seed treatment at the test site had to be achieved by alternative measures at the reference site, which were applied according to local requirements and experience at the respective farms. It was decided to conduct the early pest control during the autumn of the drilling year with pyrethroid insecticides. Since pyrethroids are non-systemic, it can be reasonably concluded that these early treatments in the year before study conduct did not influence bees which were moved onto the trial sites the following spring. The dose rate and application frequency were chosen by the farmers such that the risk posed by the increasing resistance of oilseed rape pests to pyrethroids was minimized (see Heimbach et al. 2016).

Another source of variation concerned the treatment history in both landscapes. Since they are traditional oilseed rape areas, neonicotinoid use was widespread and repeated at both the reference and test sites during the previous years. There was thus a risk that small amounts of historical neonicotinoid residues in soil could have been taken up systemically by oilseed rape plants in the fields at the reference site so that bees would have been exposed to very low residue levels as well. However, pollen and nectar from reference fields did not reveal detectable residues (see Rolke et al. 2016b). Only in processed honey, were low levels of residues detected which were, however, below the limit of quantification (1.0 µg/kg). These low levels were all well below even the lowest reported residue level which could cause effects on honey bees (e.g., comparing with the reported NOAEC for honey bees of 20 µg/kg) derived from feeding experiments using spiked diets (Schmuck and Keppler 2003). Accordingly, these levels are not considered to be relevant at least for honey bees and that the comparison of results between hives at the reference and test sites enable scientifically robust conclusions regarding the risk to bees under field exposure conditions. Consideration also needed to be given to the selection of bee species for this experiment. While domestic honey bees and bumble bees can be obtained relatively easy, the availability of native solitary bees in sufficient numbers and qualities for pesticide testing purpose is highly limited. For this reason, it was decided to use red mason bees (*Osmia bicornis*) which could be obtained in sufficient number and quality.

Accordingly, the three bee species were chosen for the study to examine not only effects to domestic honeybees but also to address the concerns raised by EFSA (EFSA 2013a) for wild bee species. The domestic honey bee (here represented by *Apis mellifera*) is considered a representative of highly social bee species exhibiting greater advanced task specialization and colony strengths of more than 50,000 individuals. The over-wintering habit requires the storage of large amounts of energy resources and, thereby, represents the worst-case in terms of duration of exposure to residues collected with either nectar or pollen. The bumble bee (here represented by *Bombus terrestris*) is considered representative for bee species with a less complex social structure but still forming colonies of considerable size, i.e., up to 500 and more individuals. The most important fitness factor for bumble bee colonies is the production rate of fertile and healthy queens which overwinter and form new colonies in the following season. Finally, the red mason bee (here represented by *Osmia bicornis*) was chosen as a surrogate species for solitary bees, which overwinter as a dormant imago inside a cocoon. Mason bees are representative for many bee species, which have not evolved social life history traits or have less complex social life history traits (e.g., *Halictus* species). The number of fertile and healthy offspring is the most important fitness parameter for this bee species.

In the study, a total of 96 queen-right, healthy colonies of *Apis mellifera* were used for both areas. There were 8 bee colonies at each of 6 study locations in both the reference and test sites. Three locations were established at the edge of oilseed rape fields to maximize the potential of bees to predominantly forage on the rape crop and so examine the effects of the upper limit of pollen and nectar, which can be collected and processed by healthy honey bee colonies under conditions of normal apicultural practice. The three other locations were established at a distance of 400 m from the nearest oilseed rape field to increase the energy consumption of the foraging bees and, thereby, to examine the effects of increased uptake of total residues from nectar of seed-treated oilseed rape plants by foraging bees as well as any effects on orientation/homing ability. Accordingly, the experimental design covered two worst-case exposure scenarios, a maximum nectar and pollen flow scenario (colonies at edge of oilseed rape fields) representing a worst-case exposure for hive bees, brood and queens and a high nectar/honey consumption rate scenario (colonies 400 m distant from oilseed rape field) representing a worst-case exposure for foraging bees.

As a representative species of bumble bees, *Bombus terrestris* was chosen. Every hive consisted of a mother queen from the same overwintering batch and 40 to 50 workers of roughly the same age. As for honey bees, bumble bee colonies were grouped into nine hives at each of six study locations (with an additional one at each location used for residue sampling). Three locations were established at the edge of an oilseed rape field whereas the other three locations were established 400 m away from the nearest rape field. The rationale of this split was the same as for honeybees, i.e., representing two worst-case exposure scenarios: maximum pollen and nectar exposure for hives adjacent to the oilseed rape fields and maximum exposure level for foraging bees of colonies placed at 400 m distance to the flowering oilseed rape crop (as well as any effects on orientation/homing ability). At the end of the study, all emerged queens and queen brood cells were counted to determine the total queen production level for each colony. It is important to note that both the hatched adult and pupal queens need to be considered since the turning point in colony development, reflecting the change from worker to reproductive offspring production, is not synchronized between colonies and can be triggered by different signals which are not necessarily related to the quality of a colony. In contrast, it can be observed that the most successful queens tend to be produced by colonies with a late-season turning point (Sterk, pers. comm.). Accordingly, studies which count only the number of hatched queens may well come to the wrong conclusions about impacts of external stressors on bumble bee colonies.

For establishing well defined populations of solitary bees at the two study sites, three nesting shelters were established at each of six locations so that they were protected against wind and rain and exposed to direct sunlight. A total of eight nesting blocks and additionally two perforated cardboard boxes containing a total number of 1500 cocoons were placed within the three shelters at each location. Again, three locations were established adjacent to an oilseed rape field and three at a distance of 100 m to the next oilseed rape field. This distance was less than for honey bees and bumblebees since mason bees are known to forage close to their nesting sites. A total of 1500 cocoons were used at each location in order to obtain a sufficient number of female individuals to achieve a population density level which allowed scientifically robust conclusions to be made on population level effects.

## Summary conclusions from large-scale monitoring study

Possible effects of clothianidin seed-treated winter oilseed rape on three different bee species (representing honey bees, bumble bees and solitary bees) were investigated in a large-scale monitoring project in Northern Germany, where oilseed rape usually comprises 25–33 % of the arable land. This study is unique in several respects:

1. The study was conducted in agricultural landscapes with multiple-year practice of intensive winter oilseed rape cultivation
2. All oilseed rape seeds in the >60 km² large reference landscape received only a commercial fungicidal seed treatment whereas all oilseed rape seeds in the respective >60 km² large treatment landscape were treated with a commercial product of the neonicotinoid insecticide clothianidin at rates recommended for insect control
3. The spatial expansion of more than 60 km² for both, the control and the treatment landscape, ensured that the introduced bee species had access only to either the control or the treated oilseed rape fields
4. During the entire bloom of oilseed rape, there were no other flowering crop plants which could have distracted bees from foraging in the oilseed rape fields. Pollen analysis though indicated that bees foraged at least temporarily on wild plants which were in flower simultaneously
5. The exposure of bees to flowering oilseed rape was maximized by drilling a combination of early and late flowering oilseed rape varieties
6. The effects of an enhanced energy demand for foraging in oilseed rape fields was investigated by placing the bee colonies in 2 different distances to the nearest oilseed rape field
7. The evaluation of all colonies of each bee species was done by the same assessment crew throughout the entire study, thus minimizing biases from evaluation by different assessors

In the study, the following endpoints were considered for the three different bee species:

**Table:** 

Endpoint assessed	Domestic honey bee (*Apis mellifera*)	Buff-tailed bumble bee (*Bombus terrestris*)	Red mason bee *Osmia bicornis*)
Colony development	X	X	n.a.
Reproductive performance	X	X	X
Pollen composition	X	X	X
Residue level in relevant matrices	X	X	X
Diseases & Parasites	X	X	X

In the case of honey bees, development of colony strength, brood success as well as honey yield and pathogen infection were unaffected with total numbers of adult bees and brood cells showing typical fluctuations and there were no significant differences between the sites. *Varroa destructor* infestation was low during most of the course of the study but increased at the end of the study due to flumethrin resistance in the mite populations but again there were no differences between the sites. In the case of bumble bees, colony development in terms of hive weight and the number of workers showed a typical course with no statistically significant differences being found between the sites. In addition, reproductive output (young queens and queen brood) cells was comparatively high and not negatively affected by the exposure to treated oilseed rape. Finally, in the case of the solitary (mason) bees overall reproductive output was high at both sites and parasitization rates remained low (less than 3 %) and within the natural range. A statistically significant higher percentage of oilseed rape pollen was collected by mason bees in the reference site compared to the test site, which may have been due to variability in oilseed rape pollen availability or the availability of alternative forage. Also, significantly more males than females were produced at the test site but the number of females did not differ significantly between test and reference sites and their mating system requires more males to be present. Accordingly, no evidence for an adverse effect on the overall fitness of populations of red mason bees was found. Overall, it can be concluded that based on the results of this large-scale monitoring study, clothianidin-dressed oilseed rape did not cause any detrimental effects on the three representative bee species.

As for the present study, also other authors concluded that neonicotinoid seed treatments in oilssed rape had no observable adverse effects on domestic honeybees. Cutler and Scott-Dupree (2007) conducted a long-term investigation to ascertain effects on honey bee, *Apis mellifera* L., colonies during and after 21 days of exposure to fields of 1-ha flowering spring oilseed rape (*Brassica napus*) grown from clothianidin-treated seed. There were four treated and four control fields, and four colonies per field, giving 32 colonies total. Residues of clothianidin found in pollen collected from bees were for most samples below the Limit of Quantification (0.5 ng/g) with peak residue levels of 2.59 ng/g. In nectar samples from randomly selected brood frames, containing stored uncapped nectar, maximum clothianidin residues of 2.24 ng/g were detected with most samples revealing residues below Limit of Quantification. The authors concluded that even the maximum clothianidin concentrations detected were 8- to 22-fold below the reported no observable adverse effects concentration. In accordance with this conclusion, no differences were observed in bee mortality, worker longevity, or brood development between control and treatment groups throughout the study. Assessment of overwintered colonies in spring also found no differences in those originally exposed to treated or control canola.

Pilling et al. (2013) conducted a 4-year field program in France to investigate any long-term effects of honey bee colonies exposed to 2 ha large flowering winter oilseed rape fields. The oilseed rape seeds had been treated with the neonicotinoid insecticide thiamethoxam at rates recommended for insect control. Median residues of thiamethoxam found in pollen collected from bees were between <1 and 3.5 mg/kg and in nectar from foraging bees were between 0.65 and 2.4 mg/kg. Median residues of the metabolite CGA322704 in pollen and nectar in the oilseed rape trials were all below the limit of quantification (1 mg/kg). Throughout the four years consecutive exposures of 12–22 days to flowering oilseed rape grown from thiamethoxam treated seeds, mortality, foraging behavior, colony strength, colony weight, brood development and food storage levels of 6 bee colonies each were similar between treatment and control colonies. Detailed examination of brood development throughout the year demonstrated that colonies exposed to the treated crop were able to successfully overwinter and had a similar health status to the control colonies in the following spring. A critical review of this study was recently conducted by Hoppe et al. (2015) who concluded limited benefit of this study since e.g. bee colonies were exposed for only short periods per year to flowering oilseed rape grown from thiamethoxam-coated seeds, a non-commercial product was used for the study at rates below the maximally allowed dosage as reported by the European Food Safety Authority (EFSA 2013b) and no statistical evaluation of the data were provided.

Cutler et al. (2014a, b) conducted another large-scale field experiment in summer 2012 in southern Ontario, Canada, to evaluate any adverse impacts of exposure to clothianidin seed-treated canola (oilseed rape) on honey bees. Colonies of honeybees were exposed to either clothianidin seed-treated or control canola fields during the 14-day bloom. There were five treated and five control fields, and four colonies per field, giving 40 colonies total. Mean residues of clothiandin found in pollen collected from the colonies were 0.84 ng/g with peak values of 1.9 ng/g. No residues were detected in nectar samples from brood frames (LOD 0.6 ng/g). Low levels of clothianidin were detected in a few pollen samples collected toward the end of the bloom from control hives, illustrating the difficulty of conducting a perfectly controlled field study with free ranging honey bees in agricultural landscapes. Overall, no effects were found of exposure to clothianidin seed-treated canola on colony weight gain, honey production, pest incidence, bee mortality, number of adults, and amount of sealed brood. Also, overwintering success did not differ significantly between treatment and control hives, and was similar to overwintering colony loss rates reported for the winter of 2012, and was similar to overwintering in Canada. The authors suggested from these results that exposure to canola grown from seed treated with clothianidin poses low risk to honey bees.

In their study investigating how neonicotinoids influence bees in real-world agricultural landscapes when applied to summer oilseed rape, Rundlöf et al. (2015) found no negative effects on honey bee colonies, which is consistent with the results from this study. However, in the case of bumble bees, they reported a negative effect on hive weights and reproduction (measured as the number of new queens). In their study though, they used non-standardized hives (unlike in this study) and the colonies were terminated at the first sight of emerging queens, which can be an incomplete way to measure reproductive performance. They also found that nesting activities of *Osmia bicornis* completely ceased on fields adjacent to oilseed rape fields treated with a clothianidin containing formulation. These results are based on a very low sample size (27 cocoons were placed at each field as compared with 1500 cocoons of *Osmia bicornis* in this study) and there was a low average emergence from these cocoons compared to the rate achieved in this study. As a result, there was a low number of exposed individuals and in such circumstances non-treatment related effects (e.g., predation and adverse weather conditions) can have a significant influence on the data. Perhaps the most important difference between the two studies, common to all the species tested, is that different crops were used. Rundlöf et al. (2015) used spring oilseed rape, which is sown in spring and flowers in the same year as it is planted, while winter oilseed rape (as used in this study) flowers in the year after it has been sown. Not only is winter oilseed rape commercially a much more important crop, it is also a much more important resource for pollinating insects due to its prolonged flowering period. Also, as the time between sowing and flowering for summer oilseed rape is shorter than in winter oilseed rape higher residue levels can be expected for summer oilseed rape. The study reported here is considered to be representative of the majority of oilseed rape cultivation areas in Europe (Heimbach et al. 2016) and reflective of the risk to both managed and wild bee species from the use of clothianidin seed-treated winter oilseed rape.
